# Supplementary material for: Characterisation of Macrophage Polarisation in Mice Infected with Ninoa Strain of Trypanosoma cruzi
Source: Pathogens. 2021 Nov 6;10(11):1444. doi: 10.3390/pathogens10111444 (PMC8622189; doi:10.3390/pathogens10111444)
Supplement: Supplementary file 1 [file pathogens-10-01444-s001.zip › pathogens-1387043-supplementary.pdf]

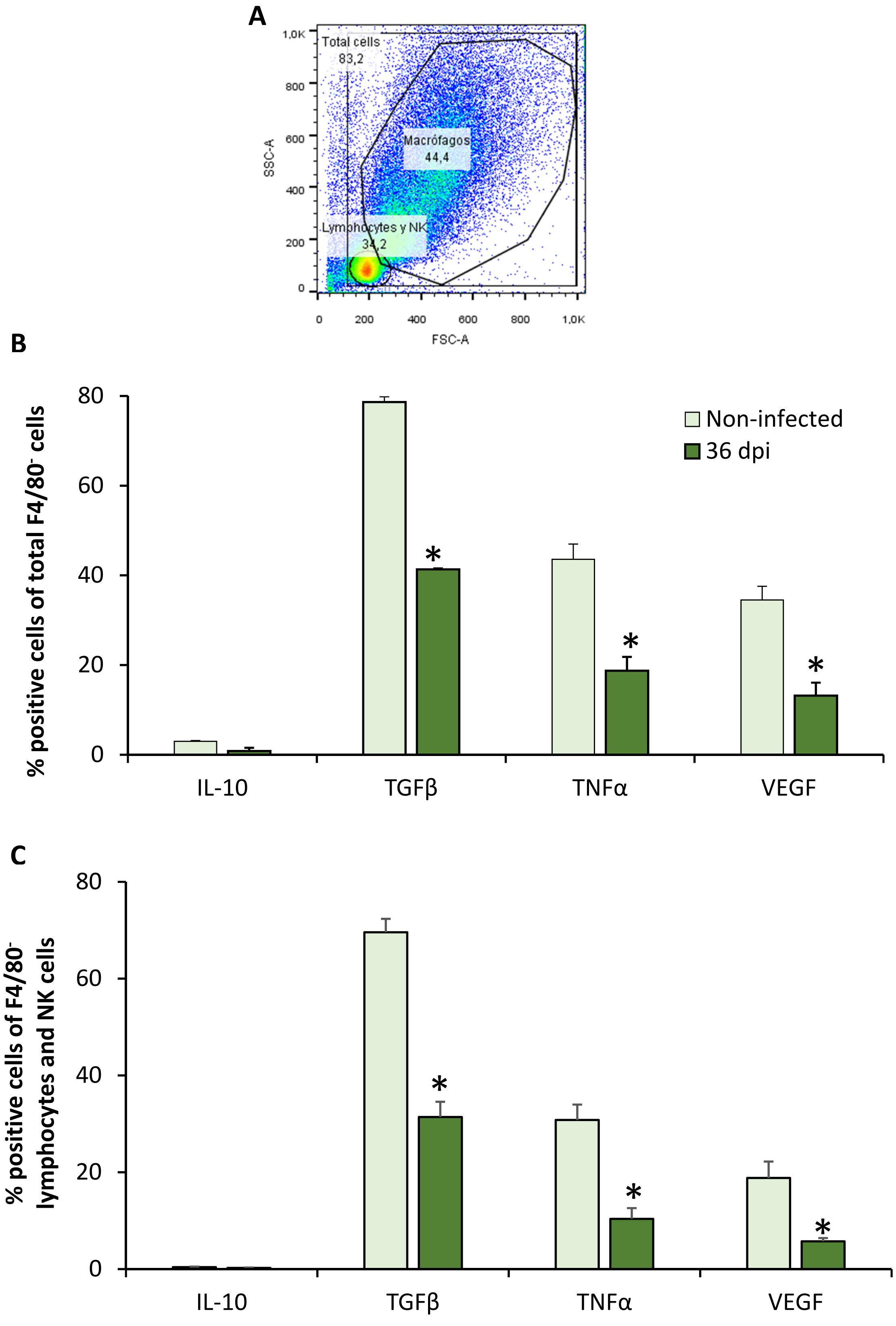

Supplementary Figure S1. Intracellular cytokines from F4/80 negative-PECs-post-infection with *T. cruzi* Ninoa strain. The C57BL/6 mice infected with 7.5 X 10^3 parasites were sacrificed at 36 dpi, and PECs were stained with specific monoclonal antibodies against F4/80 and intracellular cytokines IL-10, TGFβ, TNFα, and VEGF. The cells were analysed by flow cytometry (A) in the region of macrophages (B), and lymphocytes and NK cells (C). Non-infected mice were used as controls. Mean ± SD, n = 5. *$p < 0.05$, ANOVA post hoc Bonferroni vs. non-infected.
